# Fabrication and testing of polymer microneedles for transdermal drug delivery

**DOI:** 10.3762/bjnano.13.55

**Published:** 2022-07-08

**Authors:** Vahid Ebrahiminejad, Zahra Faraji Rad, Philip D Prewett, Graham J Davies

**Affiliations:** 1 School of Engineering, University of Southern Queensland, Springfield Central, QLD 4300, Australia; 2 Department of Mechanical Engineering, University of Birmingham, Birmingham B15 2TT, United Kingdom; 3 Oxacus Ltd., Dorchester-on-Thames, OX10 7HN, United Kingdom; 4 Faculty of Engineering, University of New South Wales, Sydney, NSW 2052, Australia; 5 College of Engineering & Physical Sciences, School of Engineering, University of Birmingham, Birmingham, B15 2TT, United Kingdom

**Keywords:** hot embossing, microneedles, penetration efficiency, thermoplastic polymers, two-photon polymerization

## Abstract

Microneedle (MN) patches have considerable potential for medical applications such as transdermal drug delivery, point-of-care diagnostics, and vaccination. These miniature microdevices should successfully pierce the skin tissues while having enough stiffness to withstand the forces imposed by penetration. Developing low-cost and simple manufacturing processes for MNs is of considerable interest. This study reports a simple fabrication process for thermoplastic MNs from cycloolefin polymers (COP) using hot embossing on polydimethylsiloxane (PDMS) soft molds. COP has gained interest due to its high molding performance and low cost. The resin master MN arrays (9 × 9) were fabricated using two-photon polymerization (TPP). A previous gap in the detailed characterization of the embossing process was investigated, showing an average of 4.99 ± 0.35% longitudinal shrinkage and 2.15 ± 0.96% lateral enlargement in the molded MN replicas. The effects of bending, buckling, and tip blunting were then examined using compression tests and also theoretically. MN array insertion performance was studied in vitro on porcine back skin using both a prototype custom-made applicator and a commercial device. An adjustable skin stretcher mechanism was designed and manufactured to address current limitations for mimicking skin in vivo conditions. Finite element analysis (FEA) was developed to simulate single MN insertion into a multilayered skin model and validated experimentally using a commercial Pen Needle as a model for the thermoplastic MNs. Margins of safety for the current MN design demonstrated its potential for transdermal drug delivery and fluid sampling. Experimental results indicated significant penetration improvements using the prototype applicator, which produced array penetration efficiencies as high as >92%, depending on the impact velocity setting.

## Introduction

During the past two decades, MN devices have become a promising tool for transdermal drug delivery, vaccination, and point-of-care diagnostics [1–2]. MNs are a painless and non-invasive method of drug delivery or sampling which can bypass the skin’s outermost layer, the stratum corneum (SC), without stimulating nerves, causing irritation, or initiating infections [2–3]. These miniature devices enable disease diagnosis and control testing beyond viruses to bacterial infections and medical emergencies, with point-of-care patch diagnostics replacing ponderous and expensive laboratory testing. Therefore, there is a growing interest in small patches incorporating mass manufacturable polymer MNs [4–5], with the point-of-care rapid diagnosis market alone predicted to grow to $50.6 billion by 2025 [6].

To enable mass manufacturing of MNs, factors such as reproducibility, fabrication precision, lower production cost, and time should be addressed. For instance, manufacturing techniques such as reactive ion etching and deep reactive ion etching incorporate multistage fabrication processes with high production costs [7]. Similarly, laser ablation and lithography techniques are costly, requiring extended production time [8]. To overcome the current manufacturing limitations, MNs might be fabricated cost-effectively, with high precision and accuracy, using 3D printing and TPP techniques [9–11]. Although additive manufacturing (AM) techniques are usually viewed as time-consuming processes, modifications and optimizations of printing parameters within the codes and algorithms of AMs can lead to significant reductions in production time [11].

MN arrays are classified into solid, hollow, coated, hydrogel-forming, and dissolvable types, which depending on the specific medical applications [12–13], are fabricated using silicon, metal, ceramic, silica glass, carbohydrate, and polymers [7,14]. In recent years, polymeric MNs have gained a lot of interest due to their biocompatibility, biodegradability, and potential for mass production [12]. Polymers such as polylactic acid (PLA), poly(methyl methacrylate) (PMMA), poly(carbonate), cyclic olefin copolymer (COC) and cycloolefin polymers (COP), polystyrene, and SU-8 photoresists, have all been used for fabrication of MNs. The low manufacturing cost and desirable mechanical properties of medical-grade thermoplastics such as COPs make them a particularly attractive choice of materials [15–16]. MN thermoplastic replicas are readily fabricated using injection molding or hot embossing [17]. However, process characteristics such as operating temperature, axial force range, and embossing time depend on material properties, geometrical size, and complexity, requiring multiple optimization studies.

MN arrays must be capable of being handled without risk of damage and must penetrate the skin with low force to the required depth [18]. There should be no MN-induced skin contamination, for example, due to breakage of the tips, and zero toxicity demands medical-grade materials. Evaluation of MN mechanical strength requires an investigation of MN insertion characteristics and possible failure scenarios. During the normal insertion of MNs, the applied force is linearly increased to the moment of rupture, which breaks the skin’s SC layer, followed by a sudden drop in the force-displacement graph [19–20]. However, the MN can be subjected to sudden excessive axial or lateral loads, which may induce early failure of the MN before skin rupture. Several methods are used to estimate these critical loads and their associated stresses, including theoretical analysis, experimental investigations, and FEA simulations [21]. For example, due to the skin’s SC barrier, the normal (vertical) insertion of MN patches on the skin may result in MN failure due to buckling. The skin’s irregular topology and inherent elasticity can also impose undesired lateral loads, resulting in transverse bending failure [14]. Prevention of possible failure scenarios can avoid MN breakage and reduce the risk of leaving residues in the skin, hence improving overall insertion safety. For the MN insertion to be mechanically safe, the safety margin (SM), which is the ratio of failure force to insertion force, should be maximized and greater than unity (SM > 1) [22].

MNs must penetrate deep enough into the skin layers to enable an effective therapeutic drug or vaccine delivery and extraction of capillary blood or interstitial fluid while avoiding stimulation of the underlying nerve system, which can cause pain to the patient [21,23]. To facilitate the penetration of the MNs, the axial force applied to the MN must be greater than the resistive force of the skin. Successful insertion is achieved upon reaching sufficient penetration depth and creating microchannels within the skin. However, the skin’s inherent elasticity and its irregular surface, with the tendency to fold around MN projections, result in unpredictable array penetration efficiency (APE), defined as the fraction of the MNs in the array passing through the stratum corneum layer without damage [24–25]. Further quantification of MN penetration is the fractional penetration length (FPL), defined as the proportion of a MN’s length penetrating the skin relative to its overall length. Meanwhile, several commercial insertion devices are patented and marketed to provide a platform for quick and pain-free insertion of MN patches, mainly for drug delivery; however, they may only be suitable for specific MN designs [26].

During MN insertion tests, the experimental setups for the measurement of FPL and APE affect the fidelity and repeatability of results [21]. To mimic the in vivo conditions of the skin, some researchers pre-stretch the sample [27–28]. But the uncontrolled initial skin strain may yield different results for otherwise similar experiments. Shu et al. recently indicated the significance of controlled skin strains on both force of insertion and MN penetration [29].

This paper investigates the reliability and fidelity of dense thermoplastic MN arrays (9 × 9) fabricated using TPP and hot embossing techniques. It considers the mechanical integrity and insertion characteristics of the arrays using theoretical, experimental, and simulation approaches. The arrays are coated with fluorescein to simulate transdermal low molecular weight drug delivery. To study MN penetration, the replicated polymer MN arrays were applied on the skin with various application methods, including dynamic impact insertion using a commercial applicator and insertion using an in-house designed and manufactured spring-loaded prototype applicator. A custom skin stretching mechanism was built to mimic skin in vivo conditions in a controlled manner. MN arrays were applied on the full-thickness porcine back skin. Pig skin possesses similarities to human skin [30]; excised dorsal (back) skin has greater stiffness compared to other skin locations [31]. The experimental results include the MN mechanical strength, mechanisms of MN damage, skin insertion force, and margin of safety prediction, along with an estimation of FPL and APE applied using different methods. The study shows the importance of custom-made impact applicators tailored for specific MN arrays to improve the APE and FPL and maintain a higher margin of safety during insertions.

## Materials and Methods

### Design and fabrication of master MN array

The MN array fabrication process uses the commercial Nanoscribe Photonic Professional GT 3D printer (Nanoscribe GmbH, Karlsruhe, Germany), providing a TPP process to make a master MN array by additive manufacturing. The 9 × 9 MN array with an overall height of 1100 µm, 250 µm base diameter, 500 µm interspacing, and 75 µm base fillet were initially designed in SolidWorks (Dassault Systems SolidWorks Corporation, Concord, NH, USA), then exported to stereolithography (STL) code.

**Figure 1:**
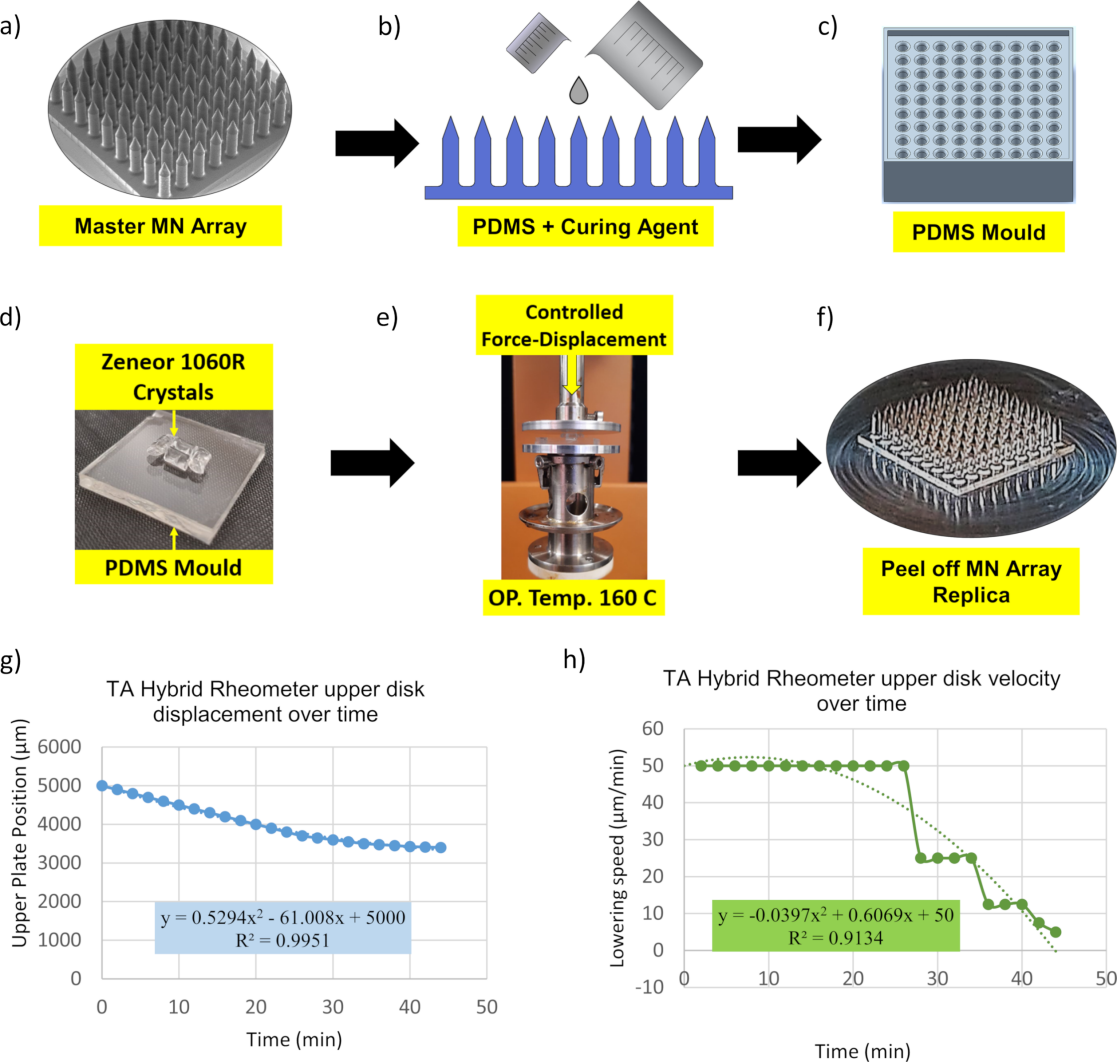
Laboratory scale process for hot embossing of COP MN array replicas using a rheometer. a) Master MN arrays fabricated using TPP technique, b) PDMS mixture solution is poured on the master mold then cured at 80 °C inside the oven, c) PDMS mold is made once master MN arrays are peeled off, d) Zeonor 1060R pellets are placed on the PDMS mold, e) hot embossing is conducted by lowering the upper disk in a controlled manner at a chamber temperature of 160 °C, f) COP thermoplastic MN array is peeled from the PDMS mold, g) the rheometer’s upper disk displacement versus time, and h) the rheometer’s upper disk speed overtime during the molding process.

The generated STL code is then imported into the DeScribe (Nanoscribe GmbH, Karlsruhe, Germany) software to adjust settings such as slicing, shell and scaffolding, laser power, and scanning speeds before converting to General Writing Language (GWL) codes. Parameters such as slicing distance of 2 µm, multiple base slide counts of 4 layers, shell and scaffolding filling method, null shear angle (0°), and laser power of 100 mW were selected after process optimization to reduce MN fabrication time and delamination from the substrate. GWL files are then imported to NanoWrite software (Nanoscribe GmbH, Karlsruhe, Germany), which is synced with NanoScribe to initiate the polymerization. The IP-S negative-tone photoresist was drop cast onto an indium tin oxide (ITO) glass substrate prior to starting the printing process. A dip-in laser lithography (DiLL) objective (25× magnification, NA = 0.8) was used for printing, after which the MN array was washed in propylene glycol methyl ether acetate (PGMEA) for 10 minutes, then rinsed in isopropanol (IPA) solution for 3 minutes. The final master MN array was carefully rinsed with deionized water and air-dried (Figure 1a).

### Manufacturing PDMS molds and MN replication

The master MNs were subsequently used to make soft polydimethylsiloxane (PDMS) molds for hot embossing the MN arrays in Zeonor 1060R COP. The PDMS solution was made by degassing the mixture of 1:10 curing agent/base ratio, which was then poured onto the master MN array and heated at 80 °C for 1 hour (Figure 1b). Samples were kept overnight to cure the PDMS mixture (Figure 1c).

To perform the hot embossing process, a rheometer (TA Instruments, New Castle, USA) was used to melt the Zeonor 1060R COP crystals, and placed in the cavities of the PDMS mold, while press forcing the sample against the mold. During this process, Zeonor 1060R crystals are placed on the PDMS mold cavities (Figure 1d), with the chamber temperature raised to 160 °C, which is 60 °C above the Zeonor 1060R’s glass transition temperature (100 °C). The rheometer is equipped with an enclosable chamber to maintain a constant temperature during the process. To perform the embossing process, the upper plate displacement and the lowering speed were set to decrease overtime to overcome the effects of viscosity that can impose abrupt pressure on the mold cavities (Figure 1e). The upper disk was lowered by ≈1.5 mm (Figure 1g) at a speed which was non-linearly reduced from ≈50 to 5 µm/min ensuring that the maximum axial force did not exceed 30 ± 2 N (Figure 1h). After embossing, the chamber temperature was set to 10 °C for 15 minutes to cool down the PDMS and thermoplastic sample and solidify the replicated microstructure. The polymeric replica of the MN array was then carefully peeled off from the PDMS mold (Figure 1f). The entire replication process for each MN array took 45 minutes.

### MN mechanical compression test

To study the failure modes of the MN arrays, a quasi-static compression test was conducted using the rheometer. A single MN with similar geometry to the 9 × 9 MN array was separately manufactured using the same process. It was assumed that the single MN projection linearly represents the 9 × 9 MN array by the factor of the number of MN projections. The single MN was attached to the lower disk of the rheometer using a double-sided tape. The upper disk was lowered with a constant velocity of 1 µm/s and traveled for 400 µm, measured from the MN tip. During the compression test, the force-displacement data were collected and plotted with MATLAB (Natick, Massachusetts, USA).

### Skin preparation and MN array insertion tests

**Figure 2:**
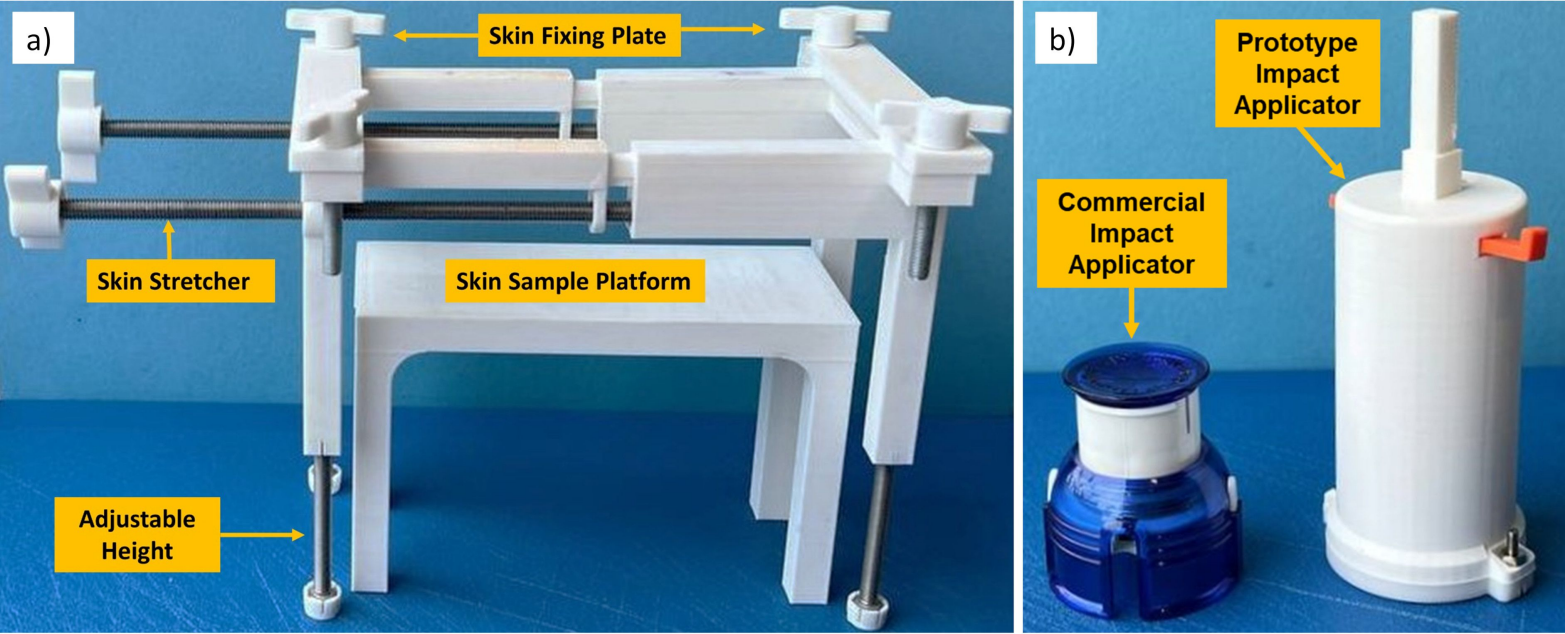
a) Skin stretching mechanism used to mimic skin condition in vivo. The skin sample is placed on the skin sample platform. Then, the stretching mechanism’s height is adjusted. Skin is then secured at both ends by fixing plates before stretching by the stretcher screws. b) A commercial applicator with a single impact speed and a prototype custom-made impact applicator capable of insertion with different impact speeds was used to apply MN arrays dynamically onto the porcine skin subjects.

Porcine back skin was used to test the penetration efficiency and insertion depth of the 9 × 9 MN arrays, using experimental procedures approved by the University of Southern Queensland (USQ) and the University of Queensland (UQ) animal ethics and biosafety committees. The skins were shaved to remove the excess hairs and kept frozen at −20 °C on a flat aluminum surface, then sectioned using a surgical knife to remove the fat layer to the thickness of 3 ± 0.1 mm [31], and thawed before insertion testing on a 3D printed stretching mechanism to mimic skin in vivo conditions (Figure 2a). The MN arrays were initially oxygen plasma cleaned for 1 minute before dip coating with a concentrated aqueous solution of fluorescein (Sigma-Aldrich Corp., St. Louis, MO, USA). Subsequently, the MN arrays were fixed onto a commercial spring-loaded applicator (Medtronic MiniMed Quick-Serter), providing an insertion velocity of 0.5 m/s. The tests were repeated using a custom-made prototype applicator, providing an insertion velocity of 1.5–4.5 m/s (Figure 2b). MN arrays were attached to the applicators’ plungers with double-sided tape and applied to the skin. The skin samples were then tape-stripped to remove the SC layer of skin before imaging.

Stereomicroscopy was then performed using a Nikon SMZ-18 microscope to determine the APE on skin subjects. The skin samples were fixed in optimal cutting temperature (OCT) compound, then sectioned to 50 µm thick slices using Leica CM3050 cryostat (Wetzlar, Germany) and placed on Superfrost glass slides. The sectioned samples were then imaged by a Zeiss LSM 710 Meta NLO confocal laser scanning microscope (Carl Zeiss, Jena, Germany) to visualize the penetration depth and estimate the FPL for individual MN projections. The images were further analyzed using ImageJ software (U. S. National Institutes of Health, Bethesda, Maryland, USA).

### Measuring the force of insertion

**Figure 3:**
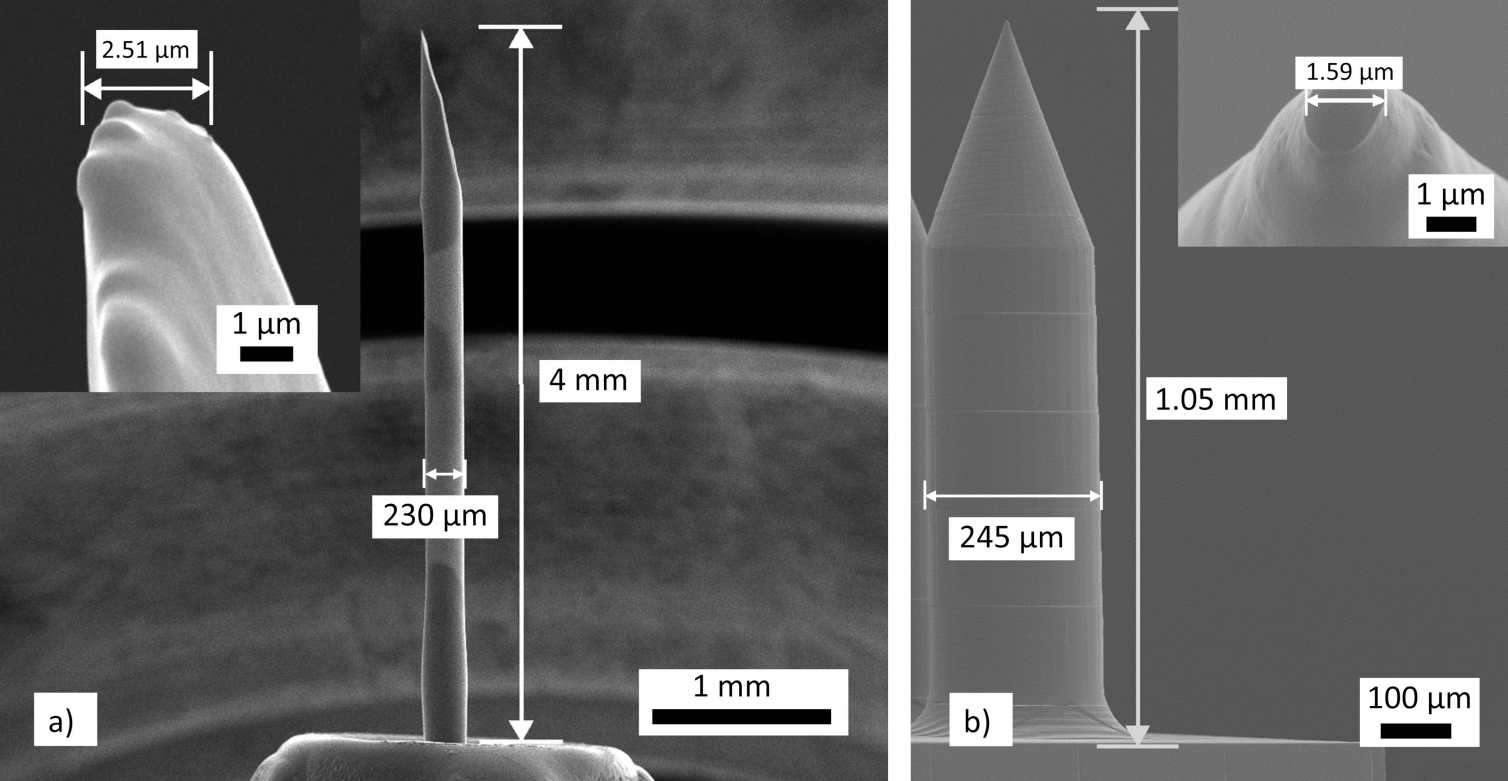
SEM images of length, tip size, and diameter of the a) BD Ultra-Fine™ 4 mm Pen Needle and b) thermoplastic Zeonor 1060R replicas.

Skin insertion tests were designed to measure the insertion force during the experiments. To facilitate the force recordings during MN insertions on porcine skin, BD Ultra-Fine™ 4 mm Pen Needles were used (Franklin Lakes, New Jersey, USA), having similar geometry to the fabricated MN array projections described above. The main reason for using PEN needles was their greater length (4 mm) which prevents early attachment of skin to the base plate, which is a common phenomenon when testing the MNs. The force of insertion is directly proportional to the square of the MN base diameter (Equation 1). Compared to other MN geometrical parameters, the dependence on the interfacial area was previously reported by Park et al. for an insertion test of polymeric MNs on human cadaver skin [22]. The representative PEN needle had a diameter of 230 µm and tip size of 2.5 µm (Figure 3a). This is similar to polymeric MNs made from Zeonor 1060R with a base diameter of 245 µm and tip size of 1.6 µm (Figure 3b). The Ultra-Fine PENs were attached to the upper disk of the rheometer using double-sided tape. The porcine back skin is fixed on the custom-made 3D printed skin stretching mechanism described above (Figure 2a) and subsequently pre-stretched to mimic the skin in vivo conditions [27]. The upper plate was lowered at 0.1 mm/s speed towards the skin while recording the force versus displacement data.

### FEA of MN insertion into the skin

To determine the MN and skin interactions during the penetration, a 2D axisymmetric simulation model was performed using ANSYS (2020 R1, ANSYS, Canonsburg, Pennsylvania, USA) Explicit Dynamics. The skin was assumed to be comprised of three layers (1) stratum corneum, (2) dermis, and (3) hypodermis with 26 μm, 2 mm, and 1.1 mm thicknesses, respectively. An Ogden (first-order) model [32] was introduced for the dermis layer, while SC and hypodermis layers were considered to possess a linear elastic mechanical response. Quadrilateral meshing with a bias factor of 5 was used to increase the number of elements in the vicinity of the skin piercing zone. Moreover, the sphere of influence meshing algorithm was used to create fine elements at the tip of the MNs.

The coefficient of friction between the contact surfaces was set to 0.42 [29]. Upon MN penetration, with a constant impact speed of 4.5 m/s, the force-displacement data were recorded to estimate the insertion force. To enable the skin piercing model, an erosion algorithm was used to eliminate the elements that reached their failure stress. To optimize the run time, the automatic mass scaling method was activated with a minimum Courant–Friedrichs–Lewy (CFL) time step of 1 picosecond [33]. The mass scale algorithm artificially increases the elemental density, which in turn reduces the overall time step by increasing the time required for a sound wave to traverse the smallest elements. Table 1 summarizes the material properties used for the individual components in the insertion simulation.

**Table 1:** Mechanical properties of different skin layers used in ANSYS Explicit Dynamics simulation.

Parameter	Microneedle	SC	Dermis	Hypodermis

mathematical model	linear elastic	linear elastic	hyperelastic: Ogden 1st order with uniaxial test data	linear elastic
thickness (mm)	n.a.	0.026	2	1.1
Young Modulus (MPa)	2100	67	n.a.	0.1
Poisson ratio	0.48	0.49	0.49	0.48
density (kg/m^3^)	1.01 E−6	1.3 E−6	1.2 E−6	9.71 E−7
hyperelastic coefficients MU1, A1 (MPa)	n.a.	n.a.	0.0568, 13.3	n.a.
incompressibility factor (1/MPa)	n.a.	n.a.	0.0745	n.a.
failure criteria (MPa)	n.a.	20	7	n.a.
Ref.	[9]	[29–30,34]	[29,35]	[29,34]

## Results and Discussions

### Design and fabrication of MN array master and replica

**Figure 4:**
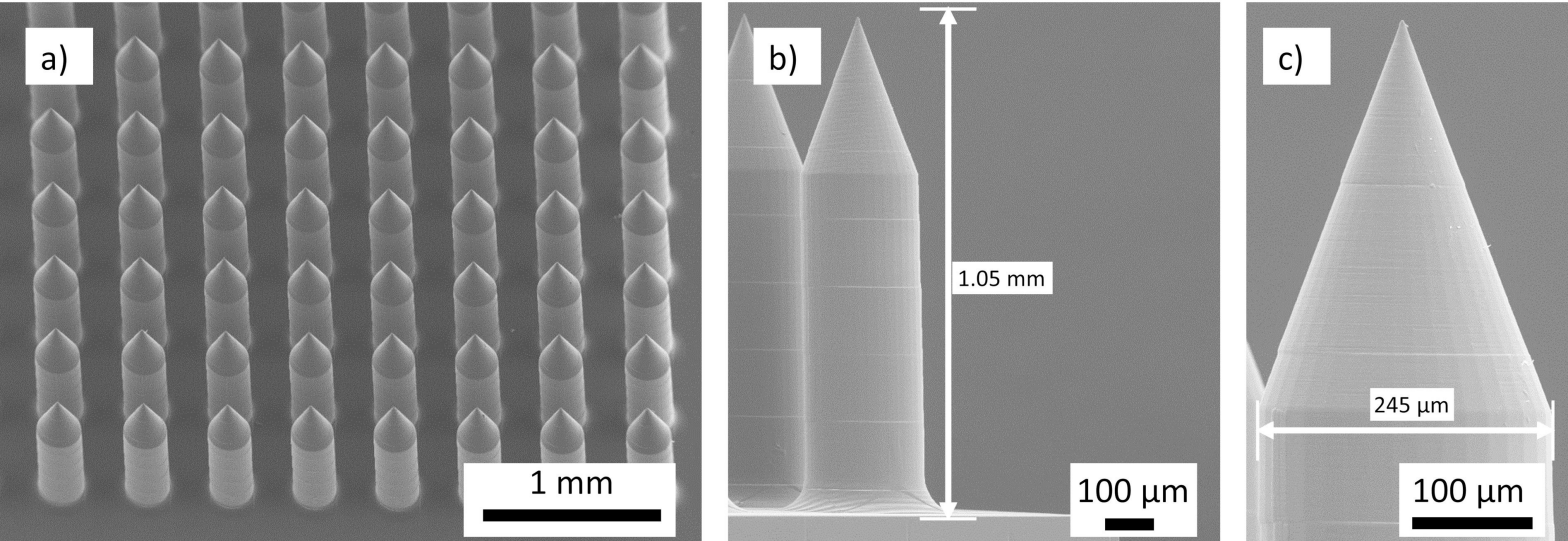
SEM of the 9 × 9 MN array, a) master MN array fabricated by TPP, b) replicated thermoplastic MN array with 1.05 mm height, and c) replicated thermoplastic MN array with a diameter of 245 µm.

The 9 × 9 MN arrays were successfully fabricated by TPP, and Zeonor 1060R replicas were made (>20 cycles) using hot embossing on PDMS mold. During the cycles, no damage was observed to the PMDS mold or its microcavities. Three 9 × 9 MN patch replicas were selected from different replication cycles of equal intervals (cycles: 1, 15, and 30). Nine projections per MN patch (*n* = 27) were selected and measured against MN master length and base diameter. The overall average length and base diameter were 1045.04 ± 3.83 µm and 255.37 ± 2.39 µm (mean ± standard deviation), respectively. The results recorded for the cycles 1, 15, and 30 indicated the respective average projection’s axial shrinkages of 4.72 ± 0.15%, 5.37 ± 0.27%, 4.9 ± 0.21% (mean ± standard deviation) (ANOVA, *p* < 0.001). Measurement for base diameters indicated enlargements of 3.22 ± 0.21%, 2.02 ± 0.33%, 1.07 ± 0.2% (mean ± standard deviation) (ANOVA, *p* < 0.001), respectively. The base diameter enlargements indicated excessive lateral forces on the cavity walls compared to longitudinal force along the axis. Figure 4a shows the SEM images of the MN array resin master, and Figure 4b,c shows the Zeonor 1060R replicas after the hot embossing, indicating slight shrinkages in both height and diameter after replication; the occurrence of small shrinkage has been reported for these thermoplastic COP materials before [9]. Thus, the effect of shrinkage needs to be considered within the initial design to ensure the dimensional accuracy of final MN replicas.

### Analytical and experimental characteristics of MN failures by mechanical compression test

Bending, buckling, and fracture are the main possible failure risks of polymer MN arrays upon insertion into the skin. Thus, investigations on the MN failure scenarios are essential and can be performed using experimental and analytical approaches. For a MN array of *N* projections to puncture the skin with the application of a vertical force *F*, the tip radius of the MNs must be small enough to exceed the puncture stress 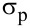. Assuming an approximately hemispherical tip, the condition on the tip radius for an array of *N* MNs applied with a force *F* is:


[1] $$r_{\text{t}}\leq \sqrt{\frac{F}{2\pi \sigma_{\text{p}}N}}\;.$$

For a particular application force (*F*), the maximum tip radius *r*_t_ can be approximated based on the skin’s ultimate stress before puncture (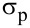).

During actual insertions, MNs are not always inserted in an exactly vertical fashion which results in lateral shear loads. This horizontal shear force component (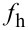) that is perpendicular to the axis of each MN may cause fracture at an approximate distance *x* from the base where the yield stress 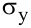 of the material is exceeded. Therefore, for MNs having a cylindrical shaft of radius *a*, with yield stress 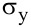 the fracture location from the base can be estimated as [36]:


[2] $$x=\frac{\pi a^{3}\sigma_{\text{y}}}{4f_{\text{h}}}\;.$$

If bending is avoided and true vertical insertion is achieved, failure may be due to buckling when the vertical force on each MN reaches the critical value (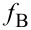) [37]:


[3] $$f_{\text{B}}=\frac{\pi^{3}a^{4}E}{16L^{2}}\;,$$

where L is the MN length, a is the MN radius, and *E* is the elastic modulus. Buckling failure load 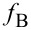 is the most important figure of merit used to determine the margin of safety of MNs.

**Figure 5:**
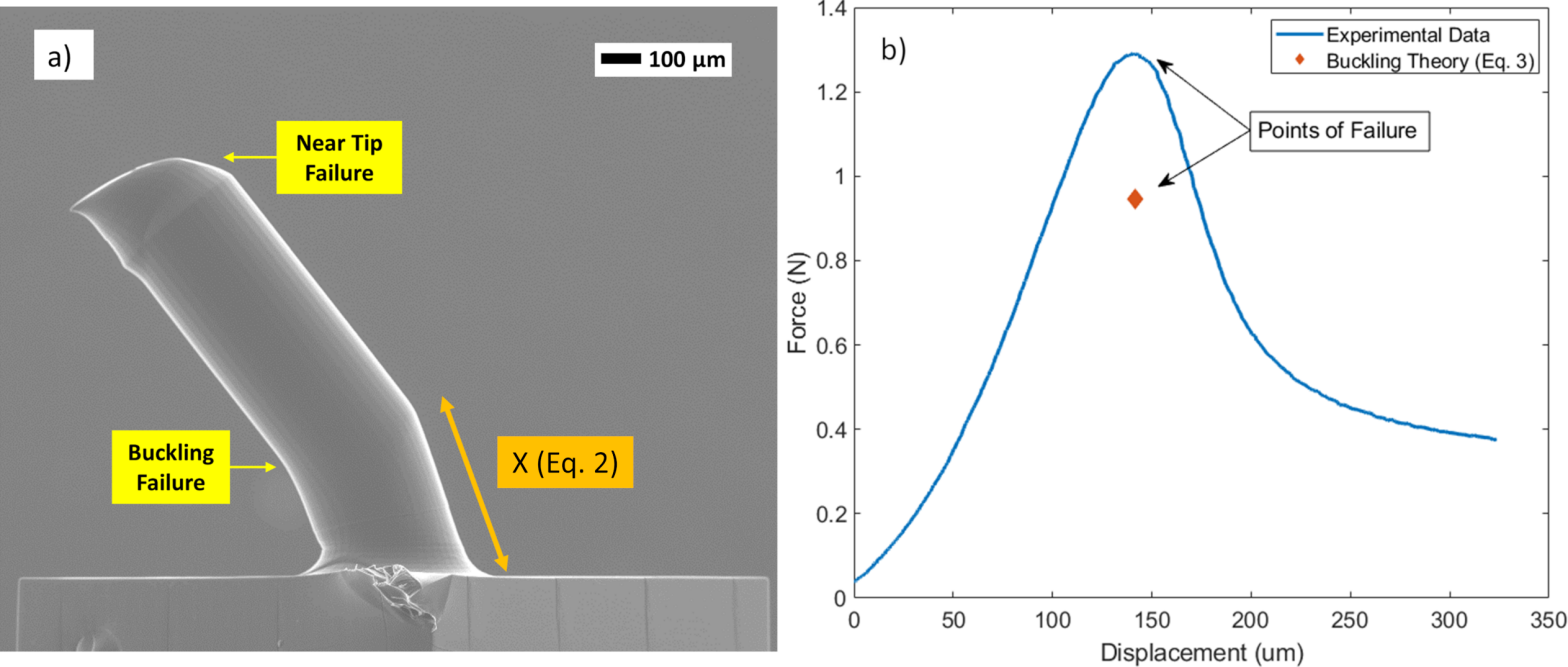
a) SEM of a MN after compression test, showing effects due to buckling and near tip failure indicating non-vertical shearing forces, b) force-displacement data for a single MN indicating a failure point of 1.26 N validated by buckling theory.

Figure 5a shows the results of mechanical quasi-static compression tests for the single replicated MN. The results revealed both near-tip yield stress failure, presumed due to the horizontal shear stress forces, and buckling failure, which occurred at the axial applied force of 1.29 N. Figure 5b illustrates the experimental force-displacement diagram for the theoretical prediction of the moment of critical buckling load. The peak on the graph indicated the MN failure. However, due to the viscoelastic nature of Zeonor 1060R, the initial near-tip failure was indistinct on the force-displacement diagram. These mechanical responses have been previously observed during compression tests on polymeric MN materials, including carboxymethyl cellulose (CMC) and polylactic acid (PLA) [1,38]. This unique viscoelastic behavior prevents the MN tip fracture, which can leave residuals in the skin. As indicated in Figure 5a, the bending location (x) and magnitude of buckling load are in alignment with Equation 2 and Equation 3. The exact location is dependent on the base diameter (125 µm), Zeonor 1060R yield stress (53 MPa), and lateral shear force component estimated during insertion. According to SEM images from samples (*n* = 3), the location of bending from the base (*x*) is at 244.4 ± 2.03 µm corresponding to a lateral shear load of 0.33 to 0.34 N. Buckling modeling was based on elastic modulus (2100 MPa) and effective penetrative length (1.025 mm) using Equation 3 and compared with experimental data. This critical buckling load was predicted to occur at 0.95 N, based on theory, whereas the experimental value was 1.29 N during compression tests. The higher value found in experimental results compared to buckling theory (Equation 3) is due to the reinforcing effects of the fillets at the MN base that improved MN stability toward sudden bending [9]. The thermoplastic Zeonor 1060R MNs had a higher failure force when compared to failure forces (0.1–0.22 N) of polylactic-*co*-glycolic acid (PLGA) MNs with a similar base diameter (200 μm) and lengths (700–1500 μm) [22].

### Simulation and experimental investigation on MN insertion force

**Figure 6:**
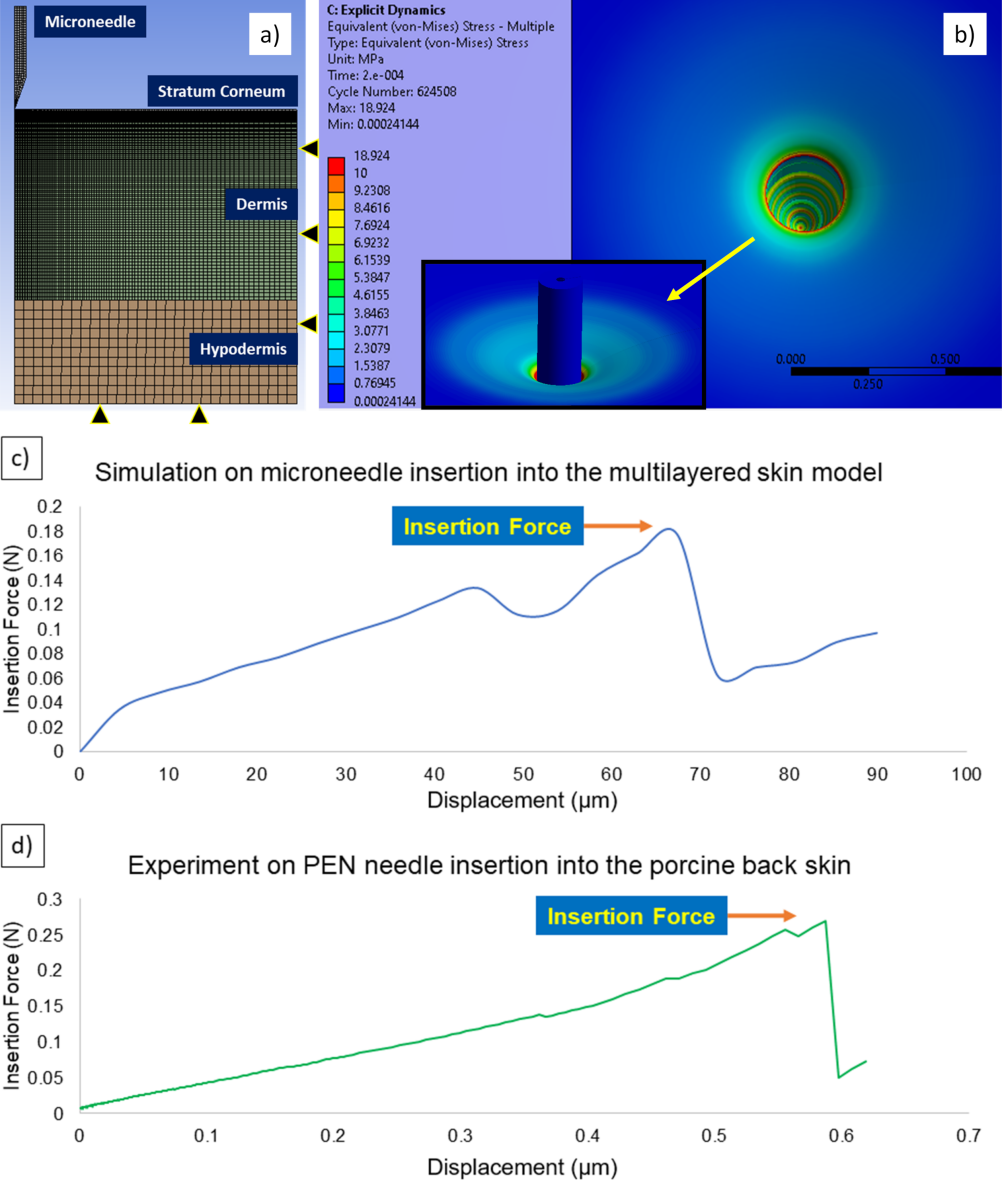
a) 2D axisymmetric meshing for three-layer skin model and single MN model, b) von-Mises stress result of the penetration region and the associated skin deflection surrounding the MN insertion. Force-displacement graph recorded during insertion based on c) FEA Explicit Dynamics simulation, and d) in vitro experiment using a BD Ultra-Fine™ 4 mm Pen Needle on porcine skin.

To investigate the insertion force and failure modes of MN arrays into the skin, the insertion of a single MN was simulated using FEA software. Figure 6a illustrates the axisymmetric model incorporating a three-layer skin model with the relevant boundary conditions. For mesh generation, the inclusion of quadrilateral elements for skin layers with a bias factor of 5 and the sphere of influence technique for MN tip yielded more accurate results due to finer meshing at the regions of MN–skin interactions. The results from the simulation showed that maximum von-Mises stress in the skin layers reached 18.9 MPa on the SC layer near the MN insertion, which is in line with the predefined failure criteria for SC and dermis layers (Figure 6b). Force displacement data were recorded and plotted during the MN insertion. The graph represented a linear increase that peaked at 0.18 N before a sudden drop due to skin fracture at the SC layer (Figure 6c).

The results were coupled with a representative in vitro experimental model using BD Ultra-Fine™ 4 mm Pen Needles. In this test, the force was linearly increased to a peak value of 0.26 N before penetrating the skin, followed by an abrupt drop in the recorded force. The ratio of buckling failure force to the insertion force was calculated using the simulation method, showing a SM of 7.16. For in vitro insertion by BD Ultra-Fine™ 4 mm Pen Needles, the SM was calculated as 4.95 (Figure 6d). The SM for both methods was above 1, indicating a sufficient safety level for skin insertions. However, SM directly depends on the MN material, its base diameter and the fillet, overall length, and the mechanics of skin subjects.

### Penetration and delivery of fluorescein into skin

**Figure 7:**
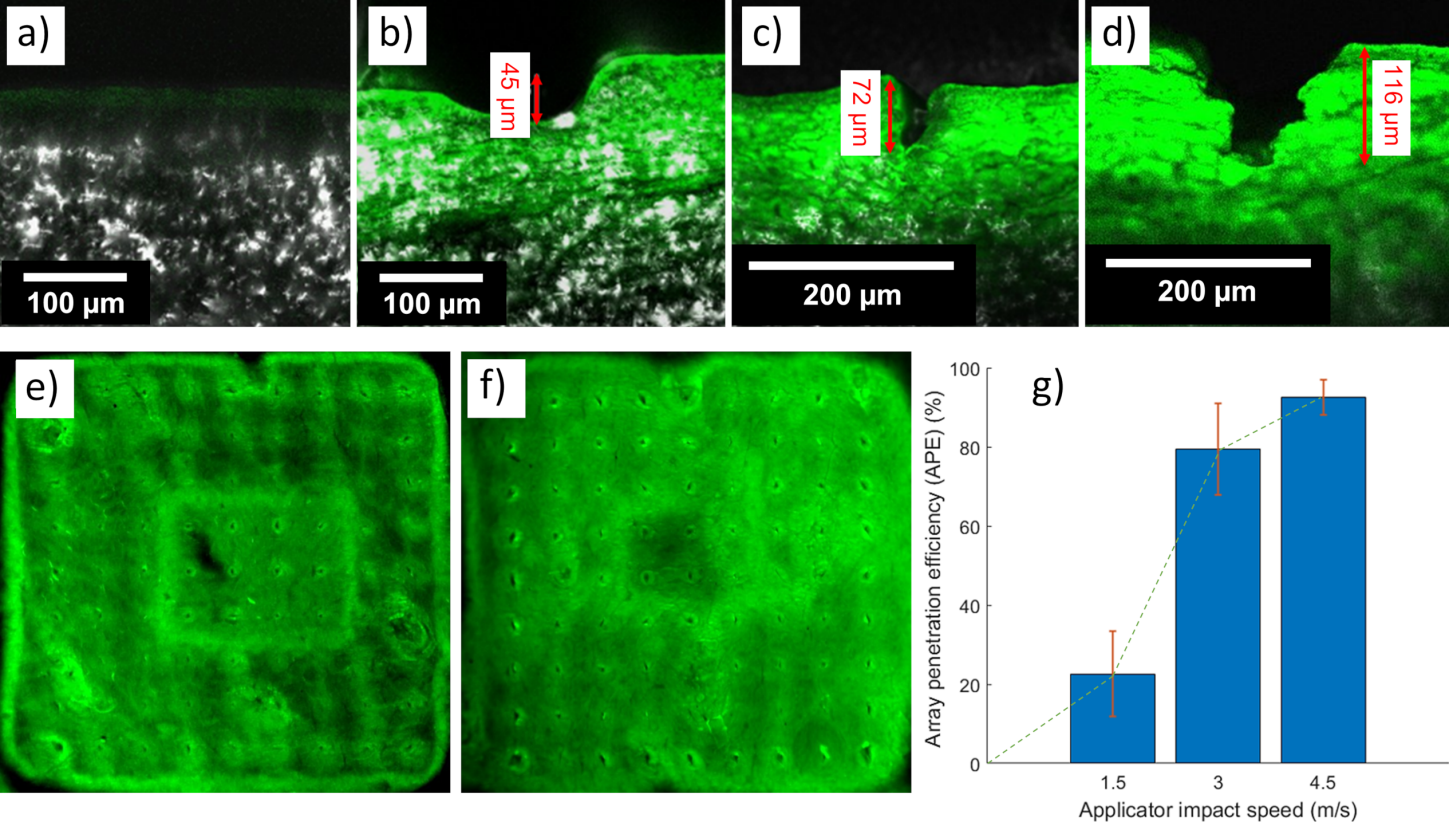
Confocal images of cryo-sectioned porcine back skin showing MN array penetration: a) control test without MNs or fluorescein solution, b) MN array patch on the skin showing no penetration using the commercial applicator, c) MN penetration and fluorescein diffusion into the skin, using the custom-made impact applicator at 3 m/s impact velocity, d) as c) for 4.5 m/s impact velocity. Stereomicroscopy images for the estimation of APE using the custom-made applicator with e) impact velocity of 3 m/s, and f) impact velocity of 4.5 m/s. g) Graph representation of the effects of the prototype applicator on APE (*n* = 3) for different impact speeds.

For MN array insertion tests on porcine skin, confocal and stereo microscopes were used to estimate the FPL and APE penetration metrics. Figure 7a–d shows confocal microscopy results of skin insertion tests for various insertion methods: (a) control, (b) using a commercial applicator, (c) using the prototype applicator at impact velocity of 3 m/s, and d) again at 4.5 m/s impact velocity. Figure 7a shows the control results where no MN array was inserted into the skin. Figure 7b indicates a skin deflection of 45 µm using the commercial applicator with 0.5 m/s impact speed revealing no penetration through the SC. In contrast, the custom applicator produced penetration of 72 µm and 116 µm (FPL of 7% and 11%) for impact velocities of 3 m/s and 4.5 m/s (Figure 6c,d). For comparison, the insertion tests of Meliga et al. on mouse ear skin produced penetration of ≈20 µm to 60 µm when their application speed increased from ≈0.25 m/s to 2 m/s [33]. In our experiments, for both applicators, insertion performance depends not only on impact velocity, but also on the number of MNs, MN interspacing, MN base diameter, and skin type, though, in this work, only impact velocity was varied. Our previous results showed that the same commercial applicator with an insertion velocity of 0.5 m/s successfully inserted a 4 × 4 thermoplastic MN patch (height: 700 µm, base diameter: 150 µm) into rabbit ear skin without deformation of the MN patch [9].

After initial penetration, APE was measured from stereomicroscopy of the diffused fluorescein patterns. There was a total failure to deliver fluorescein into the skin using the commercial applicator. In contrast, fluorescein delivery to the skin using the prototype applicator revealed APE from 22.63 ± 10.78% through 79.42 ± 11.47% to 92.52 ± 4.45% (mean ± standard deviation, *n* = 3 for each test) when the impact velocity was increased from 1.5, through 3, to 4.5 m/s (Figure 7e,f). Figure 7g shows the APE effects in bar chart form. The results show an increase of 56.79% in APE on increasing impact velocity from 1.5 to 3 m/s and an increase of 13.17% for an impact velocity increase from 3 to 4.5 m/s.

It is worth noting that Crichton et al. [39] studied the effect of varying skin strain rates on MN insertion into a rabbit’s ear. At low strain rates (≈0, 0.56, and 1.22 m/s), the APE for their Nanopatch^TM^ was as low as 25%; however, by increasing the strain rate to 5,300 s^−1^, at an insertion velocity of 1.96 m/s, an APE value of ≈95% was achieved.

The work summarized here demonstrates the potential of high-fidelity and low-cost thermoplastic MN arrays for coated drug delivery. In addition, thermoplastic MN arrays have the potential for collecting interstitial fluid more safely than using glass [40] or silicon MNs.

## Conclusion

MN arrays have considerable potential for cost-effective, rapid, and non-invasive therapeutic drug delivery, vaccination, and point-of-care diagnostics, with potential for self-administration. While large-scale manufacturing of MN arrays with high accuracy remains a challenge, the emerging technique of TPP coupled with hot embossing provides a promising, cost-effective, and highly precise method to produce batches of polymer MNs with the potential for mass production. This study fabricated Zeonor 1060R polymer MN arrays from PDMS secondary molds using a controlled hot embossing process with only minor shrinkage of the thermoplastic protrusions. The hot embossing process, tailored for current MN geometrical complexity and size, was described, including embossing time and compression speed. Key parameters were optimized to minimize the polymerization time and enhance the structural integrity during the TPP process.

A series of experiments was performed to characterize the mechanical failure and insertion characteristics of MNs: (1) axial compression test, (2) controlled insertion of BD Ultra-Fine™ 4 mm Pen Needle on porcine back skin, along with (3) Explicit Dynamics simulation of single MN insertion on a three-layered skin model. The comparisons between the results found for insertion force and quasi-static buckling test showed sufficient margins of safety (SM ≫ 1), indicating the potential of Zeonor 1060R MNs for applications in drug delivery and vaccination, with minimal associated risks. The insertion test setups for current research introduced a mechanism to enable controlled skin stretching to mimic in vivo conditions. Experiments also showed that the commercial applicator was less effective than our customized impact insertion applicator, demonstrating the need to design and manufacture customized applicators tailored for specific MN array designs.
